# Utilization of pomegranate and black grape seed by‐products in yogurt production: Effects on phenolic compounds and antioxidant activity

**DOI:** 10.1002/fsn3.3832

**Published:** 2023-12-07

**Authors:** Sibel Çalişkanlar, Derya Saygili, Nural Karagözlü, Cem Karagözlü

**Affiliations:** ^1^ Research and Application Center of Drug Development and Pharmacokinetics Ege University Bornova Izmir Turkey; ^2^ Culinary Program Izmir Kavram Vocational School Konak Izmir Turkey; ^3^ Department of Food Engineering, Faculty of Engineering Manisa Celal Bayar University Manisa Turkey; ^4^ Department of Dairy Technology, Faculty of Agriculture Ege University Bornova Izmir Turkey

**Keywords:** antioxidant, grape seed powder, polyphenol, pomegranate seed powder, yogurt

## Abstract

The present study investigated the potential utilization of pomegranate and black grape seed by‐products of the food industry in yogurt production. Specifically, we examined the effect of polyphenols on antioxidants in yogurts produced using two different starter cultures: classical yogurt culture (*Lactobacillus delbrueckii* subsp. *bulgaricus* + *Streptococcus thermophilus*) and yogurt culture supplemented with *L. casei*. Various parameters, including pH, acidity, viscosity, fat content, protein content, dry matter content, color, microbiological properties, and sensory attributes, were analyzed in the yogurt products. The present findings indicate that incorporating pomegranate and grape seed powder and using different starter cultures significantly affected the yogurt's phenolic content and antioxidant activity. Furthermore, we observed decreased phenolic content and antioxidant activity during the 21‐day storage period. Interestingly, yogurts produced with pomegranate seed powder and *L. casei* culture exhibited higher antioxidant activity than the other samples. Importantly, none of the yogurts containing pomegranate and grape seed powders had microbial counts below 10^7^ cfu/g for *L. delbrueckii* subsp. *bulgaricus*, *S. thermophilus*, and *L. casei*, indicating no adverse effects on probiotic properties. Sensory evaluation revealed that the yogurt product prepared with grape seed powder and a combination of yogurt culture (*L. delbrueckii* subsp. *bulgaricus* + *Streptococcus thermophilus*) and *L. casei* was particularly well‐received. In conclusion, the functional properties of grape seed powder and pomegranate seed powder make them suitable natural ingredients for enhancing the antioxidant activity of yogurt. The study highlights the potential of utilizing these by‐products to develop yogurt products with added health benefits.

## Graphical abstract

The search for consuming healthy products is increasing day by day and it is inevitable for consumers to turn to functional products with health‐supporting features. The enrichment of yogurt, which is a traditional product, with pomegranate and black grape seeds with high antioxidant capacity improves both the sensory properties of the final product and increases the health‐supporting quality of the final product.

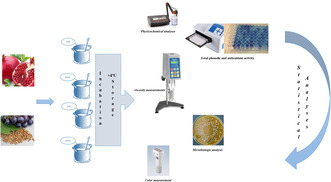

## 1 INTRODUCTION

In recent years, the demand for functional foods has increased with growing consumer awareness and desire. According to the European Commission, functional food comprises active ingredients in their natural state or foods enriched with bioactive elements or metabolites through technological processes (Anonymous, 2010).

Antioxidants can be naturally found in foods, and in the food industry, they can be added to preserve the nutritional value and maintain the quality of products. Before incorporating antioxidants into foods, it is crucial to ascertain that they do not harm human health. Natural antioxidants include vitamins (A, C, and E), polyphenolic substances, carotenoids, flavonoids, coumarins, tocopherols, and ascorbic acids (Pandey & Rizvi, 2009).

Polyphenols, or phenolic compounds, are chemical compounds characterized by hydroxyl groups attached to the benzene ring. Abundant in plants, they impart color to fruits and flowers and protect them from external factors, making them essential to human nutrition (Shrikhande, 2000). Phenolic compounds can be further classified into phenolic acids and phenolic polymers. They constitute the most important group of natural antioxidants and are commonly found in plant‐based foods such as fruits, vegetables, spices, and grains (Manach et al., 2004).

Pomegranate seeds are the pulpy part of the pomegranate that remains after processing into products like fruit juice, wine, and sauces. Approximately 10% of a pomegranate consists of seeds. The chemical composition of pomegranate seeds includes dry matter at 50.93%, protein at 37.10 g per 100 g, fat at 21.25 g per 100 g, phenolic substances at 7.20 mg per 100 g, and ash at 2.44 g per 100 g. Pomegranates are rich in phenolic compounds, such as gallic acid, ethyl brevifolin‐carboxylate, and ellagic acid, as well as triterpenes (asian acids, maslinic, ursolic, and oleanolic), flavonoids (anthocyanins and catechins), amino acids, tocopherols, sterols, and fatty acids, which together constitute 92% of its antioxidant activity (Prakash & Prakash, 2011; Özer et al., 2021).

Grapes are recognized as a source of antioxidants due to the presence of phenolic compounds and anthocyanins. Grape seeds, a by‐product obtained during grape processing, are particularly rich in polyphenols (phenolic compounds). Grape seeds contain approximately 7% phenolic substances, including flavonoids and phenolic acids (Monagas et al., 2003).

Secondary products derived from food and agricultural residues, such as bark, seeds, stems, straw, and leaves, are believed to benefit human health due to their antioxidative properties. This research investigates the effect of various yogurt cultures on the antioxidant activity of yogurts manufactured by incorporating pomegranate seeds and grape seed powder supplemented with polyphenol extracts.

## 2 MATERIALS AND METHODS

### 2.1 Material

Raw cow's milk (pH 6.74 ± 0.07, Solid Nonfat 8.12 ± 0.08%, fat 3.15 ± 0.16%, protein 3.06 ± 0.01%, ash 0.29 ± 0.08%, and lactic acid 0.13 ± 0.01%) was sourced from the Department of Animal Science at Ege University. Freeze‐dried yogurt cultures (YoFlex) and a single strain of *Lactobacillus casei* 431 cultures were obtained from Chr. Hansen in Copenhagen, Denmark. Grape (*Vitis vinifera*) seed powder (GSP) was procured from Tazetemiz Co. in Mersin, Turkey. Pomegranate (*Punica granatum* L.) seed powder (PSP) was supplied by Mecitefendi Co. in Izmir, Turkey.

### 2.2 Methods

#### 2.2.1 Preparation of yogurts

For yogurt production, the no‐fat dry matter of raw cow's milk samples, taken at +4°C, was standardized to 12% using milk powder. According to the literature reviews (Akca & Akpinar, 2021; Altunkaya et al., 2013; Bagchi et al., 2002; Baydar et al., 2004; Bchir et al., 2012, 2019; Chouchouli et al., 2013; Demirbüker Kavak & Akdeniz, 2019; Demirkol & Tarakci, 2018), pomegranate seed powder (1%) and grape seed powder (0.5%) were added. Subsequently, the mixture underwent thermal treatment for 10 min at 85°C and was then cooled to the incubation temperature. Each product was inoculated with a distinct yogurt culture. The samples were incubated at 42 ± 0.1°C for yogurt cultures and 40 ± 0.1°C for yogurt cultures and *L. casei* until a pH of 4.6 ± 0.1 was achieved. After incubation, the samples were refrigerated at 4 ± 1°C, and the analyses on the 1st day were conducted 24 h later. On the 1st day, analyses covered fat, dry matter, ash, protein, and antioxidant content. pH, acidity, microbiological, sensory, viscosity, and sensory analyses were carried out on the 1st, 7th, 14th, and 21st days. Antioxidant content and color analyses were conducted on the 1st and 21st days. Production processes were executed at the Ege University Dairy Technology Pilot Plant, and storage was maintained at 4 ± 1°C. The experimental design of yogurts is given in Table 1.

**TABLE 1:** Experimental design of yogurts.

Samples	GYC	GYCC	PYC	PYCC
Grape (*Vitis vinifera*) seed powder	1%	1%	–	–
Pomegranate (*Punica granatum* L.) seed powder	–	–	0.5%	0.5%
*Lactobacillus delbrueckii* subsp. *bulgaricus* + *Streptococcus thermophilus* cultures (YK)	3%	–	3%	–
*Lactobacillus delbrueckii* subsp. *bulgaricus* + *Streptococcus thermophilus*, and *Lactobacillus casei* cultures (YKC)	–	3%	–	3%

*Note*: GYC: Yogurt made from grape seed powder and yogurt culture; GYCC: Yogurt made from grape seed powder, yogurt culture, and *L. casei*; PYC: Yogurt made from pomegranate seed powder and yogurt culture; PYCC: Yogurt made from pomegranate seed powder, yogurt culture, and *L. casei*.

#### 2.2.2 Physicochemical analyses

In yogurt and milk, the alkali titration method, as outlined in AOAC (2003), was employed to determine specific parameters. A digital pH meter (Hanna pH 211 Microprocessor, Portugal) was utilized to measure the pH levels of yogurt and milk. Additionally, the total dry matter, fat, and protein content were assessed following the procedures outlined in AOAC (2003).

#### 2.2.3 Apparent viscosity measurements

The evaluation of yogurt sample viscosity was conducted by means of a Brookfield DV‐II + Pro Model viscometer (Middleboro, USA), which was fitted with an LV4 spindle and operated at a rotational speed of 120 rpm. This assessment involved the determination of torque values spanning from 10% to 90%, following the methodology outlined by Saygili et al. (2021). The data were captured through the utilization of RHEOCALC 32 Application Software, and the resultant viscosity measurements were expressed in units of millipascal‐seconds (mPa·s).

### 2.3 Total phenolic and antioxidant activity of the yogurts

The total polyphenols were quantified in yogurt samples following the procedure outlined by Beskow et al. in 2015. The Folin–Ciocalteu reagent was combined with distilled water in a 1:1 ratio to initiate the assay. Subsequently, each yogurt sample was thoroughly mixed using a vortexer. The reaction blend was supplemented with sodium carbonate (Na_2_CO_3_) and left to incubate at ambient temperature for a period of 60 min. Following this incubation, the absorbance was quantified at a wavelength of 725 nm utilizing a Thermoscientific Multiskan Sky spectrophotometer situated in the United States. Additionally, a blank sample reading was obtained at the same wavelength. The total phenolic content was ascertained through a calibration curve, expressed by the equation *y* = 1.45597 + 0.0249791*x*, which exhibited a high coefficient of determination (*R* ^2^ = .999). The results were reported in milligrams of gallic acid equivalents (GAE) per milliliter (mg GAE/mL).

To evaluate the radical scavenging properties against DPPH, we adopted the methodology originally devised by Brand‐Williams et al. in 1995, making slight modifications as necessary. Specifically, we combined 100 μM of DPPH solution dissolved in methanol with an equal volume of 100 μL of yogurt samples within a 96‐well plate, thus achieving a 1:1 ratio. Following this, the resultant mixture was incubated under dark conditions at room temperature for a duration of 30 min. Subsequently, the absorbance was measured at 517 nm. A blank solution was prepared, omitting the extract, and methanol was employed to calibrate the spectrophotometer (Thermoscientific Multiskan Sky, USA). The antioxidant activity was quantified as the percentage of inhibition of the DPPH radical and computed using the following formula:

$$\%\;\mathrm{RSA}=\left[\left({\mathrm{Abs}}_{\text{control}}–{\mathrm{Abs}}_{\text{samples}}\right)/{\mathrm{Abs}}_{control}\right]\times 100$$

Evaluating ABTS (2,2‐azino‐di‐(3‐ethylbenzothialozine sulfonic acid)) radical scavenging activity followed the protocol outlined in Re et al.'s work from 1999. Specifically, a 10 μL aliquot of the diluted sample was combined with 240 μL of reagent and allowed to undergo a 10‐min reaction at 25°C. Subsequently, the absorbance was quantified at 734 nm using a 96‐well Microplate Reader (Thermo Fisher 1530, Finland). The outcomes were expressed as μmol/L of Trolox equivalent antioxidant capacity, abbreviated as TEAC.

#### 2.3.1 Color measurement

In the assessment of yogurt samples, color attributes were quantified using a Minolta Meter CR 400 (Konica Minolta, Inc., Osaka, Japan). Specifically, the L* parameter measured luminance or lightness, the a* parameter determined the degree of redness or greenness, and the b* parameter quantified the extent of yellowness or blueness in each yogurt sample. These measurements were conducted in triplicate for each yogurt sample, following the methodology outlined by Pan et al. (2019).

#### 2.3.2 Microbiologic analysis

A 10‐g sample of yogurt was mixed thoroughly with 90 mL of sterile Ringer's solution and subsequently diluted as per the recommended dilution ratios. To quantify the population of *S. thermophilus*, the pour‐plate technique was employed on M17 agar. Following inoculation, the Petri dishes were placed in an incubator set at 37°C for a duration of 48 h. Enumeration of *L. delbrueckii* subsp. *bulgaricus* was conducted using MRS agar medium within anaerobic jars supplemented with Anaerocult A (Merck, Germany) and incubated at 42°C for 72 h, following the procedure as outlined by Donkor et al. (2006). For quantifying *L. casei*, plating was performed on MRS‐V agar and incubated at 37°C for 72 h under anaerobic conditions, following the methodology described by Tharmaraj and Shah (2003).

#### 2.3.3 Statistical analyses

The current investigation utilized SPSS software version 20.00 (SPSS Inc., Chicago, IL, USA) for the evaluation of the impact of four different yogurt formulations and storage conditions on a range of parameters, encompassing pH, acidity, dry matter content, protein content, fat content, viscosity, probiotic viability, color measurements, total phenolic content, and total antioxidant capacity. A significance threshold of *p* < .05 was employed for detecting variations. To discern noteworthy differences among the results, one‐way ANOVA and Duncan's Multiple Range Test were applied. The experiments were performed in duplicate, with each analysis executed using three replicates.

## 3 RESULTS AND DISCUSSION

### 3.1 Physicochemical properties

Statistical analysis revealed that the differences in fat, ash, and protein values among the yogurts were statistically not significant (*p* > .05). While the PYCC sample exhibited the highest values, the GY sample had the lowest solid nonfat (SNF) content. This suggests that including pomegranate seed powder (1%) and grape seed powder (0.5%) in yogurt production may affect SNF content. The SNF content in the yogurt samples aligns with findings from prior studies, indicating higher SNF values compared to plain yogurts (Bchir et al., 2019; Bolek, 2020; Kowaleski et al., 2020; Sahingil & Hayaloglu, 2023; Sendra et al., 2010).

In accordance with Table 2, the titratable acidity levels (represented by lactic acid) exhibited fluctuations ranging from 0.96 to 1.21 throughout storage, with a statistically significant increase in lactic acid content observed as storage time progressed (*p* < .05). According to the guidelines established by the WHO/FAO Codex Alimentarius Commission in 2011 (FAO, 2013), a minimum titratable acidity level of 0.6% is required for yogurt to meet the standard requirements.

**TABLE 2:** Proximate composition of yogurt samples.

	SNF (%)	Ash %	Fat %	Protein %
GYC	11.73 ± 1.13^a^	0.89 ± 0.10	2.86 ± 0.43	4.62 ± 0.19
GYCC	12.20 ± 1.67^a^	1.09 ± 0.57	2.90 ± 0.98	4.72 ± 0.20
PYC	12.77 ± 1.30^b^	0.94 ± 0.92	2.80 ± 0.28	4.64 ± 0.25
PYCC	13.61 ± 0.14^b^	0.97 ± 0.94	2.60 ± 1.97	4.85 ± 0.36

*Note*: (a, b) Different letters in the same column represent the difference at the *p* < .05 level.

**FIGURE 1:**
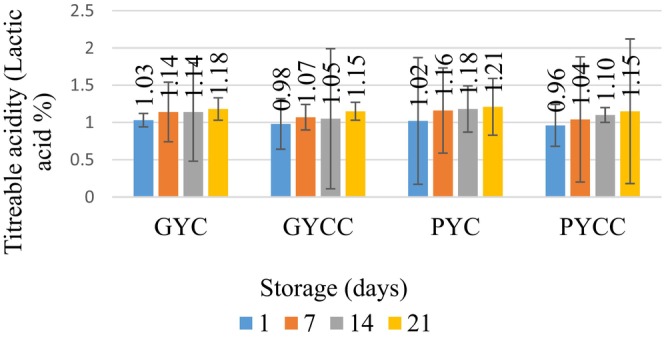
Titratable acidity (lactic acid) % changes in yogurt during storage.

The titratable acidity rate of yogurts made with the *L. casei* culture was lower than that of those made with the classical yogurt culture. *L. casei* culture is believed to reduce acidity, possibly due to the more extended fermentation period or slower development. The variation in acidity values between yogurts with pomegranate seed and grape seed powder may be attributed to the differing acid content of grape and pomegranate seeds. The acidity of yogurt samples made with black grape seed powder ranged from 0.85% to 0.99% (Figure 1). It is suggested that changes in the acidity and pH values of the samples are affected by the acid content of the grape seed and lactic acid fermentation (Kalyas & Ürkek, 2020). It has been determined that an increase in acidity is vital for product stability (Deshwal et al., 2021).

**FIGURE 2:**
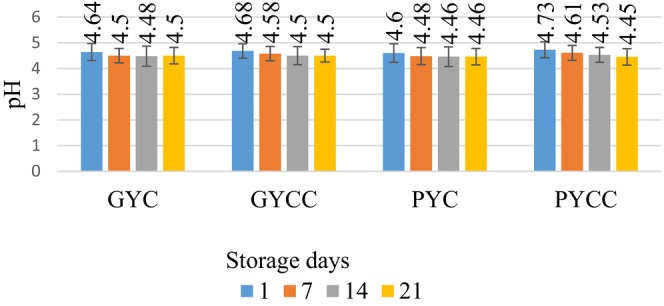
Variation of the pH value throughout the study period.

As shown in Figure 2, the storage duration of yogurt products is characterized by pH values ranging between 4.45 and 4.73. A statistically significant decrease (*p* < .05) in pH values was observed across the samples during storage. Different strains of lactic acid bacteria (LAB) exhibit varying levels of pH tolerance. For example, *S. thermophilus* demonstrates resilience and can thrive at pH levels as high as 5, while other strains, such as *L. delbrueckii* subsp. *bulgaricus* and *L. casei*, can withstand pH levels as low as 4 (Deshwal et al., 2021). The addition of pomegranate and grape seed powder extracts did not have a discernible effect on the pH values of the outcomes (*p* > .05). The preservation process is the primary factor contributing to pH reduction, where lactose is broken down into lactic acid. An increase in these acids leads to decreased pH values during fermentation (Ranasinghe & Perera, 2016). Consequently, the findings indicate that the incorporation of various extracts into yogurt did not have an adverse effect on the pH values of the samples. In line with studies conducted by El‐Samh et al. (2013), Yadav et al. (2018), Bchir et al. (2019), and Ferreira and Santos (2023), which investigated the factors responsible for pH reduction in yogurt enriched with pomegranate and grape seed powder, similar results were obtained. Hence, the results collectively suggest that including phenolic extracts from food and agricultural by‐products in yogurt is feasible without compromising pH levels, ensuring that the product remains suitable for consumption.

**FIGURE 3:**
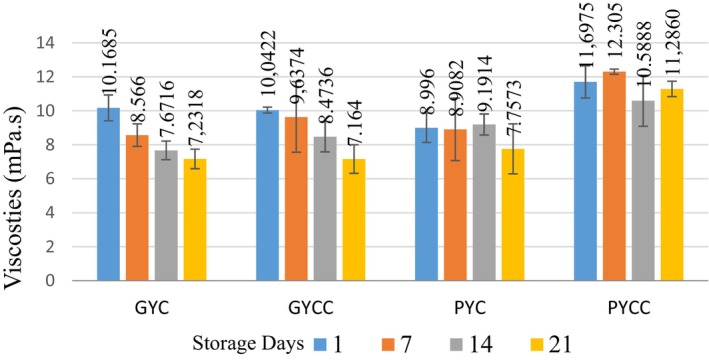
Viscosity of yogurt products made from grape and pomegranate seed powder (mPa·s).

The viscosity characteristics of yogurt were affected by incorporating pomegranate and grape seed powder, as illustrated in Figure 3, which displays the viscosity trends. The viscosity of the yogurt was notably affected and was found to be statistically significant (*p* < .05). This effect was observed concerning the type of pomegranate and grape seed powder used and the duration of storage. On the seventh day, a significant decrease in yogurt viscosity was observed, after which it remained relatively stable during the storage period. This decrease in viscosity can be attributed to the combined effect of both pomegranate and grape seed powder, as well as the specific yogurt cultures employed. Notably, it has been ascertained that black grape seed powder boasts a high fiber content of 71% (Kurt, 2015). Previous studies have also reported a favorable effect of fiber addition on preventing serum separation in yogurts (Ozcan & Kurtuldu, 2014). A study on yogurts enriched with dietary fiber established that viscosity values increased with higher fiber content (Bakirci et al., 2017).

### 3.2 Total phenolic content (TPC) and antioxidant capacity

The research assessed the effect of integrating phenolic extracts derived from various residual materials into yogurt formulations. Table 3 presents the Total Phenolic Content (TPC) and antioxidant capacity measurements for yogurts enriched with pomegranate and grape seed powder. The TPC of yogurts made with pomegranate seed powder is higher than that of samples made with grape seed powder. It was observed that using different yogurt cultures effectively influenced the TPC of yogurts (*p* < .05). Similarly, a statistically significant decrease in the TPC during storage was observed (*p* < .05).

**TABLE 3:** Total phenolic content and antioxidant capacity of pomegranate and grape seed powders used in the production of yogurts.

	Day	GYC	GYCC	PYC	PYCC
Total phenolic content (mg GAE/g)	1	3.84 ± 0.55^aX^	4.04 ± 0.42^aY^	2.56 ± 0.21^aX^	2.91 ± 0.35^bY^
	21	3.14 ± 0.54^b^	3.24 ± 1.05^b^	2.46 ± 0.18^aX^	2.79 ± 0.19^bY^
D.P.P.H. (%)	1	68.10 ± 6.51^a^	70.21 ± 4.51^a^	65.71 ± 2.36	70.62 ± 0.41^a^
	21	59.39 ± 12.02^b^	60.04 ± 10.33^b^	63.54 ± 0.55	63.63 ± 3.15^b^
T.E.A.C. (μM Torolox/g)	1	0.5279 ± 1.96	0.4906 ± 1.84	0.5703 ± 2.16^a^	0.5446 ± 2.06^a^
	21	0.5191 ± 2.02	0.4816 ± 1.86	0.5294 ± 2.08^b^	0.5170 ± 1.91^b^

*Note*: (a, b) (↓): Different letters in the same column represent the difference at the *p* < .05 level. (X, Y) (→): Different letters on the same line indicate a difference at the *p* < .05 level.

In plain yogurts, the source of total phenolic substances includes low‐molecular antioxidant compounds, free amino acids, and peptides. The total phenolic content (TPC) in plain yogurt typically falls within the range of 0.84–1.30 mg GAE/g (Akan, 2022; Helal & Tagliazucchi, 2018). Notably, the addition of pomegranate and grape seed powder, rich sources of polyphenols, has a positive effect on increasing the phenolic content of yogurt. These findings align with the results of previous studies by Tseng and Zhao (2013), Chouchouli et al. (2013), Karaaslan et al. (2011), Ersoz et al. (2011), and Bolek (2020). Furthermore, several studies have reported that using probiotic bacteria in yogurt culture increases TPC levels (Amirdivani & Baba, 2015; Ferreira & Santos, 2023). This effect is attributed to the enhanced breakdown of milk proteins by probiotic bacteria (Damin et al., 2009; Shah, 2000).

Over 21 days, all examined samples consistently showed decreased ABTS and DPPH contents. This trend is in line with findings reported by Chouchouli et al. (2013), Karaaslan et al. (2011), Ersoz et al. (2011), Bchir et al. (2019), and Sahingil and Hayaloglu (2023). However, it was observed that the choice of distinct yogurt cultures significantly affected (*p* < .05) the levels of DPPH and ABTS in yogurt products. Variations in the data may arise from differences in sample preparation (Lehtinen & Laakso, 1997), extraction methods (Zieliński & Kozłowska, 2000), and hydrolysis procedures (Nuutila et al., 2002), leading to discrepancies in antioxidant activity determination methods. The deliberate selection of the DPPH compound in the present study was based on its solubility exclusively in organic solvents, its effective oxidative capabilities, and its role as both an oxidizing substrate and a reaction indicator (Sah et al., 2014).

The DPPH and ABTS values in plain yogurts vary between 42.63% and 60.22% and 0.56 and 7.07 mg Trolox/100 g in yogurts produced with *L. delbrueckii* ssp. *bulgaricus*, *S. thermophilus*, and *L. acidophilus* cultures (Akan, 2022). In yogurts produced with *L. acidophilus*, *B. bifidus*, and *S. thermophilus*, the DPPH value has been reported as 54.2% (Demirbüker Kavak & Akdeniz, 2019), and DPPH content varies between 19.01% and 20.64% in yogurts produced with ABT 7 culture (Elaltunkara, 2018). In a study of the antioxidant activity of different starter cultures using the DPPH method, *L. casei* culture exhibited 78.5% antioxidant activity, *L. brevis* showed 81.9%, and *L. delbrueckii* ssp. *bulgaricus* strain had 56.3% (Ural & Yüksekdağ, 2020). The antioxidant capacity in plain yogurt is derived from various bioactive peptides obtained from milk proteins through the proteolysis of LAB (Gómez‐Ruiz et al., 2008).

Previous studies have consistently demonstrated a positive correlation between the overall phenolic content of polyphenolic extracts from fruits and their ability to scavenge free radicals, as documented by Skrede et al. (2004) and Caillet et al. (2006). The present study's results align with these findings, as supported by Chouchouli et al. (2013), Karaaslan et al. (2011), Ersoz et al. (2011), and Ferreira and Santos (2023). These findings suggest that including pomegranate and grape seed powder in yogurt formulations enhances the yogurt's antioxidant properties, and different starter cultures also affect the antioxidant activity. Additionally, in line with similar investigations, we observed a decline in antioxidant activity during the storage period, as indicated by Yadav et al. (2018).

### 3.3 Color analysis

The study measured the colorimetric values (L*, a*, and b*) of the yogurt samples, as detailed in Table 4. Consumer acceptance of a food product is closely linked to its visual appeal and color, both of which serve as critical indicators of product quality. Typically, color is the primary sensory characteristic that consumers notice, and it has the potential to affect other sensory perceptions, including aroma and taste, significantly. The color of yogurt is susceptible to changes due to various factors, such as shelf life, storage duration, and discoloration, as discussed by Coggins et al. (2010).

**TABLE 4:** Hunter Lab color values of the yogurt containing pomegranate and grape seed powder.

	GYC	GYCC	PYC	PYCC
*L*				
1	71.75 ± 2.29	72.71 ± 4.2	74.72 ± 1.77	69.80 ± 7.70
21	74.62 ± 3.59	72.65 ± 2.26	72.60 ± 2.74	68.02 ± 4.82
*a*				
1	0.04 ± 0.29^aY^	0.08 ± 0.1^aY^	0.23 ± 0.04^aX^	0.20 ± 0.18^aX^
21	0.47 ± 0.2^bY^	−0.05 ± 0.14^bZ^	0.43 ± 0.07^bY^	0.86 ± 0.1^bX^
*b*				
1	6.91 ± 0.27^Y^	7.11 ± 0.4^Y^	9.76 ± 0.41^X^	8.28 ± 0.79^X^
21	6.98 ± 050^Y^	7.72 ± 0.2^Y^	9.47 ± 0.19^X^	8.71 ± 0.49^X^

*Note*: (a, b) (↓): Different letters in the same column represent the difference at the *p* < .05 level. (X–Z) (→): Different letters on the same line indicate a difference at the *p* < .05 level.

The L* (whiteness) value of yogurts with PSP and GSP added is between 74.72 and 68.02. The change in L* value during the storage period and using different seed powders in yogurts was also not statistically significant (*p* > .05). It was determined that the whiteness of the yogurts decreased when pomegranate and grape seed powder were added.

According to the Hunter scale, a value represents red (−) and green (+). The a* value was found in the range of 0.86 to −0.05. On Day 21, the value of GYCC yogurt was negative. The differences between storage days and yogurt varieties were statistically significant (*p* < .05). It is thought that anthocyanin in pomegranate and grape seeds may be effective in causing this decrease (Bchir et al., 2012; Małgorzata et al., 2016).

The b* (yellow‐blue) value of the yogurt samples did not change during the storage period and was found to be statistically insignificant (*p* > .05). However, the b value of yogurts with pomegranate seed powder added was higher than in yogurts with grape seed powder added, and this difference was found to be statistically significant (*p* < .05). This difference may be attributed to the presence of grape oil in the grape seed powder. This variation may be due to the grape seed powder's grape oil.

The present L*, a*, and b* results are consistent with those reported by Tarakçı (2010), Damian (2013), Arjöamd (2011), Chouchouli et al. (2013), Demirkol and Tarakci (2018), Elaltunkara (2018), Yadav et al. (2018), Akca and Akpinar (2021), and Sahingil and Hayaloglu (2023).

### 3.4 Microbiological characteristics

**FIGURE 4:**
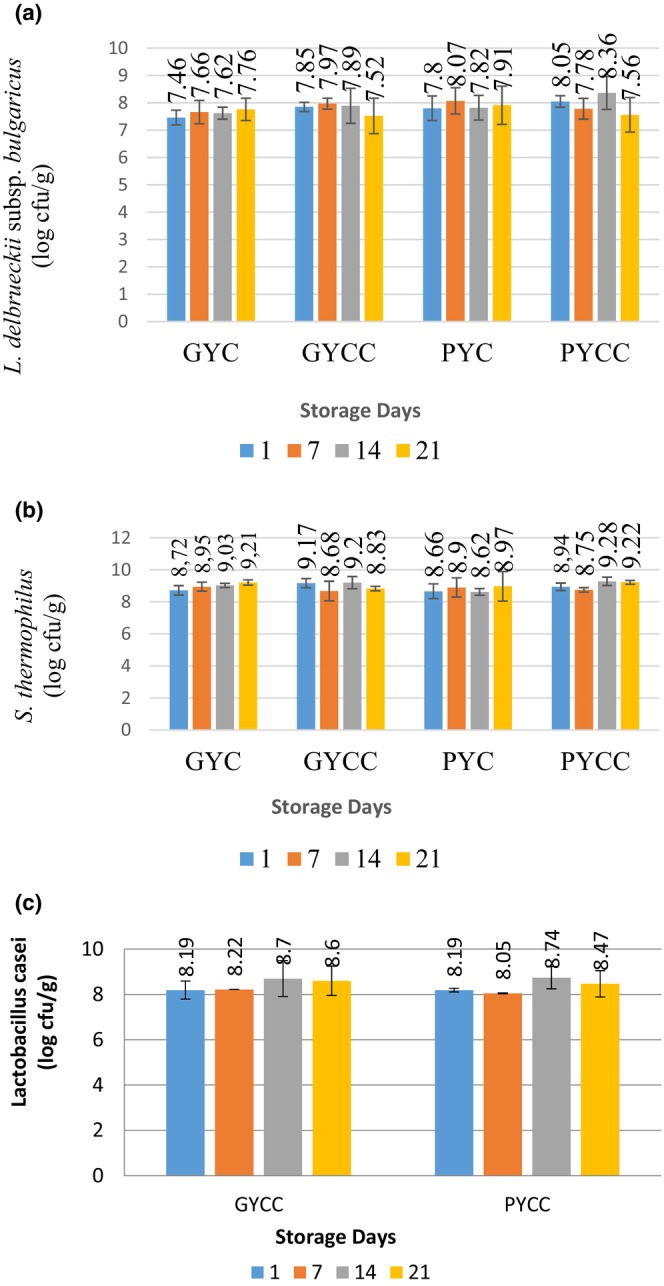
*Lactobacillus delbrueckii* subsp. *bulgaricus* (a), *Streptococcus thermophilus* (b), and *L. casei* (c) counts (log CFU/g) of yogurt products containing grape and pomegranate seed powder.

In order to confer health benefits to humans, it is imperative that live cultures retain their activity throughout their designated shelf life (Deshwal et al., 2021). Consequently, an investigation was carried out to assess the microbiological attributes of yogurts enriched with grape and pomegranate seed powder over the course of their storage duration. The quantification of *L. delbrueckii* subsp. *bulgaricus*, expressed as log cfu/g, did not exhibit any statistically significant distinctions between yogurts enriched with grape seed and those enriched with pomegranate seed powder (*p* > .05). Similarly, there was no notable divergence in the log cfu/g counts of *L. delbrueckii* subsp. *bulgaricus* throughout the storage period (*p* > .05). It is worth noting that, by the end of the storage period, the log cfu/g count of *L. delbrueckii* subsp. *bulgaricus* witnessed an increase in the case of grape yogurt culture (GYC) and pomegranate yogurt culture (PYC) samples. In contrast, it decreased for samples of grape yogurt control culture (GYCC) and pomegranate yogurt control culture (PYCC) (refer to Figure 4). This phenomenon is thought to be influenced by the presence of the *L. casei* culture. The rise in the count of *L. delbrueckii* subsp. *bulgaricus* at the conclusion of the storage period may be attributed to heightened titration acidity during storage, dissolved oxygen levels, and bacterial characteristics (Hamann & Marth, 1984; Salvador & Fiszman, 2004). Previous research has proposed that the upsurge in *L. delbrueckii* subsp. *bulgaricus* at the end of storage can be ascribed to the bacterium's resilience to acidic environments and low pH levels (Beal et al., 1989).

The *L. delbrueckii* subsp. *bulgaricus* counts were consistent with the results of previous studies (Baydar et al., 2004; Chouchouli et al., 2013; Ersoz et al., 2011; Yadav et al., 2018). The count of *L. delbrueckii* subsp. *bulgaricus* remains above 10^7^ cfu/g, indicating that pomegranate and grape seed powder do not significantly affect the microorganism count during yogurt production.

A significant change in the *S. thermophilus* counts (log cfu/g) during storage was observed (*p* < .05). By the end of storage, the count of *S. thermophilus* log cfu/g increased in GYC, PYC, and PYCC samples but decreased in GYCC and PYCC samples, likely due to the presence of *L. casei* culture (Figure 4). The present results align with Ersoz et al. (2011), Chouchouli et al. (2013), Yadav et al. (2018), and Atwaa et al. (2022). Additionally, phenolic compounds in pomegranate and grape seeds are believed to contribute to the increase in *S. thermophilus* count. Previous studies have shown that the addition of herbal extracts, rich in phenolic compounds, fibers, and organic acids, enhances the activity and growth of LAB (Etxeberria et al., 2013; Nicolesco & Buruleanu, 2010; Tanaka et al., 2016; Yoon et al., 2005).

The difference in *L. casei* counts between yogurts enriched with grape seed powder and pomegranate seed powder was statistically significant (*p* < .05) (Figure 4). The present findings corroborate the results of studies by Abdel‐Hamid et al. (2020) and Kowaleski et al. (2020). However, there was no significant change in the count of *L. casei* during storage (*p* > .05). Similar trends were observed in the yogurts of Güler‐Akın et al. (2016) and Elaltunkara (2018), who used *L. acidophilus* as a probiotic in yogurt production. In contrast, the microorganism count decreased during the storage period in the yogurts of El‐Samh et al. (2013), which employed *B. lactis* Bb‐12.

### 3.5 Sensory evaluation

The differences in appearance and color values between yogurts enriched with pomegranate and grape seed powder were insignificant (*p* > .05). In contrast, significant differences were observed in consistency, flavor, and odor values (*p* < .05). Significant differences were noted in the appearance, consistency, flavor, and odor of yogurts enriched with pomegranate and grape seed powder during storage (*p* < .05).

**FIGURE 5:**
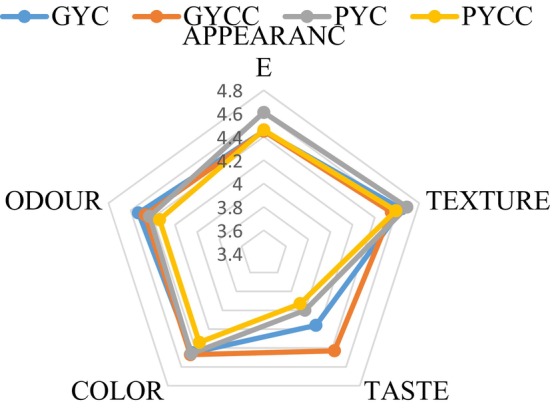
Sensory evaluation of yogurt products made from grape and pomegranate seed powder.

Upon evaluating the sensory analysis results of the yogurt samples, it was determined that, in terms of appearance and consistency, the PYC sample excelled. In contrast, the GYCC sample received the highest scores for flavor, color, and odor (see Figure 5). This preference for the GYCC sample is believed to be attributed to the inclusion of grape seed powder and *L. casei* culture.

## 4 CONCLUSION

The significance of functional foods and probiotics has surged in recent years, mainly due to compelling evidence of their positive effect on human health, leading to increased consumer demand. Industrialization and environmental pollution have substantially elevated the presence of free radicals, contributing to the onset of numerous chronic diseases. Adopting a natural, well‐balanced, and mindful diet is imperative to mitigate these diseases. Consuming foods rich in antioxidants has been shown to mitigate the deleterious effects of harmful free radicals. Consequently, the present study aimed to substitute synthetic antioxidants, typically added to foods as preservatives, with natural antioxidants comprising various polyphenolic compounds with the same antioxidative properties.

This research revealed that food industry by‐products such as pomegranate and grape seed powder, along with starter cultures, positively affected yogurt's phenolic and antioxidant characteristics. Particularly in terms of flavor, yogurt produced with grape seed powder and *L. casei* culture garnered higher consumer preferences. Pomegranate and grape seed powder are readily available at a low cost, offering ample potential for developing diverse fermented dairy products. Therefore, producing cost‐effective, functional dairy items incorporating pomegranate and grape seed powder is feasible.

## AUTHOR CONTRIBUTIONS

**Sibel Çalişkanlar:** Methodology; Validation; Formal analysis; Investigation; Writing—review & editing. **Derya Saygili:** Methodology; Validation; Formal analysis; Investigation; Writing—review & editing; Writing—original draft; Conceptualization. **Nural Karagözlü:** Methodology; Investigation; Validation; Formal analysis; Writing—review & editing; Conceptualization. **Cem Karagözlü:** Methodology; Investigation; Validation; Methodology; Formal analysis; Writing—review & editing; Conceptualization.

## Data Availability

The data that support the findings of this study are available from the corresponding author upon reasonable request.
